# *Strongyloides stercoralis* prevalence and diagnostic efficacy of an IgG4 rapid test in an eosinophilic population in Khuzestan Province, southwestern Iran

**DOI:** 10.1186/s13071-025-06910-z

**Published:** 2025-07-22

**Authors:** Alireza Ashiri, Abdollah Rafiei, Rahmah Noordin, Nor Suhada Anuar, Ali Teimoori, Bijan Ansari-Moghaddam, Molouk Beiromvand

**Affiliations:** 1 Cellular and Molecular Research Center, Medical Basic Sciences Research Institute, Ahvaz Jundishapur University of Medical Sciences, Ahvaz, Iran; 2 Department of Parasitology, School of Medicine, Ahvaz Jundishapur University of Medical Sciences, Ahvaz, Iran; 3 Infectious and Tropical Diseases Research Center, Health Research Institute, Ahvaz Jundishapur University of Medical Sciences, Ahvaz, Iran; 4 Department of Parasitology and Medical Entomology, Faculty of Medicine, Universiti Kebangsaan Malaysia, 56000 Kuala Lumpur, Malaysia; 5 Institute for Research in Molecular Medicine, Universiti Sains Malaysia, 11800 Penang, Malaysia; 6 Department of Virology, Faculty of Medicine, Hamadan University of Medical Sciences, Hamadan, Iran; 7 Department of Immunology, Lorestan University of Medical Sciences, Khorramabad, Iran

**Keywords:** Strongyloidiasis, Eosinophilia, Khuzestan Province, Iran, Prevalence, Coprological methods, *Strongyloides* IgG4 rapid test (IgG4 RDT), ELISA

## Abstract

**Background:**

*Strongyloides stercoralis* is a pathogenic nematode affecting the human intestine. Chronic strongyloidiasis often remains asymptomatic, posing diagnostic challenges due to the low sensitivity of conventional methods. Using traditional methods, this study investigated the prevalence of strongyloidiasis in Khuzestan Province, southwestern Iran. We also studied the effectiveness of a *Strongyloides* immunoglobulin G4 (IgG4) rapid diagnostic test (RDT) for timely infection detection before and after treatment.

**Methods:**

This cross-sectional study, conducted during 2022–2024, evaluated 520 participants with eosinophilia (> 5%) for *S. stercoralis* infection. Coprological methods used were direct smear stool microscopy and agar plate culture. Serological methods were enzyme-linked immunosorbent assay (ELISA) (NovaTec^®^ kit) and a prototype IgG4 RDT using a recombinant antigen (NIE) . Traditional coprology and composite references were used to assess the diagnostic power. Among copro-positive patients, 30 cases were followed up at least 3 months after treatment using the same methods.

**Results:**

Of the 373 participants who submitted stool samples, coprological methods identified 95 positive cases, with culture proving to be more sensitive than direct smear (24.9%, 93/373 versus 7.5%, 28/373). Of the 520 participants, 35.2% (183/520) and 43.7% (227/520) tested positive for *S. stercoralis* using ELISA and IgG4 RDT, respectively. Spearman’s rank correlation between the IgG4 RDT and ELISA was significant (*ρ* = 0.772; *P* < 0.001). Despite minor discrepancies, the IgG4 RDT showed substantial agreement with the ELISA (*κ* = 0.776). Increased eosinophil counts were strongly associated with *Strongyloides* infection with a mean of 20.48% in copro-positives versus 15.22 in copro-negatives and area under the curve (AUC) of 0.741 and 0.701 for coprology and the combination of coprology and serology methods (CRS), respectively. In the 30 follow-up patients, a significant reduction in eosinophil counts (*P* < 0.001) was observed. Five cases (17%) remained larva-positive, and serological tests significantly increased readings/scores. Three copro-negative patients showed strong positive results on ELISA and IgG4 RDT.

**Conclusions:**

On the basis of the obtained results, the prevalence of *S. stercoralis* infection among the eosinophilic population was high. This study showed that the IgG4 RDT is a reliable and efficient diagnostic tool for *S. stercoralis* infection. The rapid test results demonstrated significant agreement with the ELISA and effectively detected infection in eosinophilic patients, making it a suitable diagnostic test for screening, particularly in resource-limited settings.

**Graphical Abstract:**

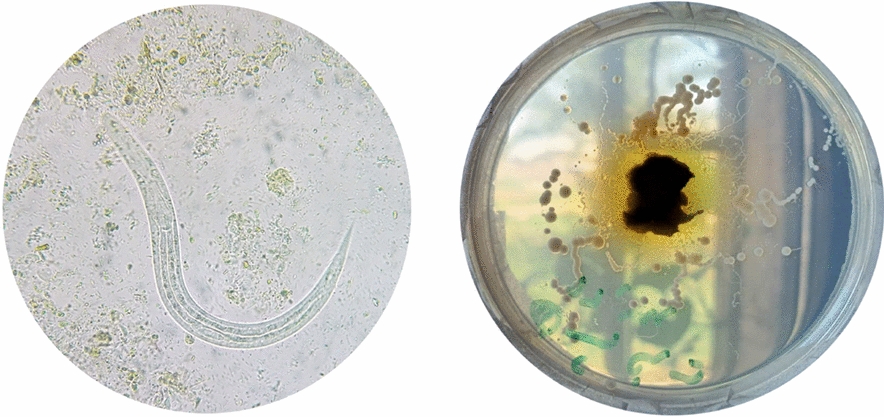

**Supplementary Information:**

The online version contains supplementary material available at 10.1186/s13071-025-06910-z.

## Background

*Strongyloides stercoralis* (*S. stercoralis*), a pathogenic nematode affecting the human intestine, is a facultative parasite with a unique and complex life cycle. This parasite is recognized as a significant contributor to neglected tropical diseases [1]. Recent estimates suggest that approximately 600 million people globally might be infected, with Iran classified as a low-prevalence area, reporting less than 5% infection [2]. The interplay between the potential for autoinfection and the host’s immune status enables this parasitic infection to manifest in two distinct forms: uncomplicated (chronic strongyloidiasis) and complicated (hyperinfection syndrome or disseminated form). In its uncomplicated forms, strongyloidiasis faces diagnostic challenges due to the low sensitivity of conventional diagnostic methods. In contrast, the complicated forms pose clinical challenges, including septicemia, pneumonia, and meningitis, which can lead to high mortality rates [3].

Chronic strongyloidiasis, the most prevalent infection form, often remains asymptomatic and clinically silent [4]; such cases are often compared to a ticking time bomb or silent assassin owing to the risk of progressing to severe infection stages [5, 6]. Persistent and unexplained eosinophilia often serves as the sole indicator that raises suspicion of *Strongyloides* infection in these individuals. However, identifying these hidden cases is one of the most challenging aspects of its diagnosis, primarily owing to the limited sensitivity of routine diagnostic techniques [7, 8].

While there is consensus on the importance of timely screening, diagnosis, and treatment, the primary challenge is the lack of an error-free diagnostic method, commonly called the “gold standard” [9]. Existing diagnostic methods show variations in the trade-off between sensitivity and specificity. Traditional techniques, such as direct smear, formalin–ether concentration, the Baermann technique, and agar plate culture (APC), demonstrate good specificity; however, their sensitivity is often inadequate owing to low and intermittent larvae excretion. Furthermore, these methods are time-consuming, labor-intensive, dependent on technician skill, and often require serial sampling, which is impractical for large-scale epidemiological studies [1, 10]. Nucleic acid amplification methods lack standardized extraction and amplification protocols, leading to variable results. Additionally, the sensitivity of these techniques, similar to coprological methods, depends on the larval load excreted, rendering them not viable for use in underprivileged and endemic areas owing to the high costs of specialized equipment and the reliance on skilled professionals [11].

Serological methods, despite some limitations, are effective tools for identifying cryptic cases of strongyloidiasis. Among them, the enzyme-linked immunosorbent assay (ELISA) stands out for its high sensitivity. However, its specificity may be affected by cross-reactivity [12]. Fortunately, using recombinant antigens, such as NIE and *Strongyloides stercoralis* immunoreactive antigen (SsIR), rather than somatic parasite antigens has enhanced the specificity of this technique [13, 14]. Reformatting ELISA into a point-of-care test (POCT) that meets the Affordable, Sensitive, Specific, User-friendly, Rapid and robust, Equipment–free, and Deliverable to end user (ASSURED) criteria would facilitate timely identification of infected individuals in field studies as well as individual diagnoses [15]. In light of the inclusion of strongyloidiasis in the world Health Organization (WHO) roadmap for controlling neglected tropical diseases by 2030, the relevant guidelines emphasize the importance of further research, recommending the development of faster and more sensitive diagnostic methods while also calling for comparative studies of existing techniques [16, 17].

In this study, we estimated the prevalence of *Strongyloides* infection among patients with eosinophilia and associated risk factors in Khuzestan Province, one of the least studied regions in southwestern Iran. We also compared the diagnostic efficiency of coprological and serological techniques (direct smear, APC, ELISA, and *Strongyloides* IgG4 RDT) before and after treatment.

## Methods

### Study design, area, and sampling

This cross-sectional study assessed the coprological and serological prevalence of strongyloidiasis among eosinophilic patients referred to the diagnostic and care centers south of Khuzestan Province during 2022–2024. This province is in the southwest of Iran (31° 20′ N, 48° 40′ E), covering an area of 63,238 km^2^ with a population of 4,936,000. Its main economic activities include agriculture, industry, trade, and fishing. According to a previous study, Khuzestan is recognized as one of the areas endemic for *S. stercoralis* [18].

### Sample size

On the basis of the 42% prevalence reported in a previous study conducted in a similar population in northern Iran [19], the minimum sample size was calculated using Cochran’s formula for single population proportion. With parameters *P* = 0.42, *d* = 0.05, and *α* = 0.05, the required sample size was 374. To ensure robustness and account for potential exclusions, 520 eligible volunteers were recruited. However, 146 participants did not provide stool samples, and thus, only serological tests were performed for these individual.

### Sample collection and processing

Participants were referred from outpatient clinics and hospitals in the area between 2022 and 2024 after being referred for documented relative eosinophilia (> 5%) in previous evaluations. A confirmatory complete blood count (CBC) was performed using Mindray BC6200 or Sysmex X11800 analyzers. Cell differentiation results were validated through smear preparation and Giemsa staining to determine the absolute eosinophil count (standard clinical threshold). Notably, 34 patients with relatively high eosinophilia had a normal absolute eosinophil count of fewer than 500 cells per microliter of blood, yet they were included in the study. This decision was based on the understanding that relative eosinophilia, even at lower absolute counts, could provide valuable insights into underlying conditions and variations in eosinophil behavior. Inclusion was random and independent of symptoms or comorbidities, reflecting the real-world heterogeneity of the patient population. Individuals under 5 years old, those who had consumed antiparasitic medications within the preceding 6 months, and nonindigenous individuals were excluded from the study.

All participants completed a questionnaire with demographic information (age, sex, place of residence, occupation, and education level) and clinical details (underlying conditions and infection-related symptoms).

After centrifugation and separation, participants’ serum samples were transferred to the Department of Parasitology at Ahvaz Jundishapur University of Medical Sciences and stored at –70 °C until serological tests were conducted. Fresh stool samples were examined through coprological examinations at the Infectious Disease Laboratory of 17 Shahrivar Hospital, Abadan County. Among the 30 patients followed up post-treatment (with timing dependent on patient cooperation), 8 retrospective cases from our earlier records (2018–2021) with confirmed strongyloidiasis and post-treatment follow-up data were included to enhance longitudinal diagnostic analysis.

### Statistical analysis

Data analysis was performed using SPSS 19 (SPSS Inc., Chicago, IL, USA) and GraphPad Prism (GraphPad Software, Inc., San Diego, CA, USA). Descriptive statistics were employed to summarize the demographic, laboratory, and clinical characteristics of participants. Continuous variables are expressed as mean ± standard deviation (SD), while categorical data are reported as absolute frequency and percentage (*n*/*N*, %). Diagnostic performance metrics (sensitivity, specificity) were calculated with their 95% confidence intervals (CI) to ensure robust clinical interpretation. The diagnostic performance of the techniques was assessed by comparing them against the traditional coprological reference and composite reference standards, using receiver operating characteristic (ROC) curve analysis to calculate the area under the curve (AUC). The ROC curves for ELISA and IgG4 RDT display a stepped pattern owing to the binary nature of their outcomes (positive/negative), as opposed to producing continuous probability scores [20]. The agreement among the techniques was evaluated using Cohen’s kappa coefficient (*κ*). The Mann–Whitney *U* test was used to compare groups, while the Wilcoxon signed-rank test was applied for within-group comparisons of eosinophil count before and after treatment. Additionally, Spearman’s rank correlation coefficient was calculated to determine the relationship between the intensity of reactions in the serological methods. All statistical analyses were conducted with a significance level set at *α* = 0.05.

### Coprological tests

#### Direct stool examination (DS)

A homogeneous suspension of approximately 2 mg of fresh stool was prepared in a droplet of normal saline (0.9%) on a clean glass slide and thoroughly examined under a light microscope at 100× and 400× magnifications by experienced parasitologists [21]. Distinguishing features of *S. stercoralis* larvae are the short buccal cavity, straight gut, a rhabditiform esophagus (divided into three sections) extending one-third of the body length, and lateral rhomboid genital primordium halfway down the larval body [22, 23].

#### Agar plate culture (APC)

Nutrient agar culture medium (Ibresco life Science, lot no. NA210331008) was prepared, sterilized, and poured into Petri dishes. Approximately 3 g of stool sample was placed in the center of an agar plate and incubated for 1 week at 25 °C. The plate was sealed with cellulose tape to mitigate potential risks of environmental contamination. From the second day, it was examined macroscopically and microscopically for any signs of larval movement. The final diagnosis was confirmed by washing the surface of the agar with 10% formalin solution, centrifuging, and then microscopically examining the sediment for larvae or adult worms [24].

### Serological tests

#### Enzyme-linked immunosorbent assay (ELISA)

ELISA was conducted using the NovaLisa^®^ *Strongyloides* antibody kit (NovaTec Immunodiagnostica GmbH, Dietzenbach, Germany) and Dynex DS2^®^ automated ELISA analyzer (Dynex Technologies, USA). The kit is designed to detect total IgG and IgM immunoglobulins against *Strongyloides* spp. by employing a sandwich ELISA that utilizes a chimeric recombinant antigen and A/G conjugated protein. The test was performed according to the manufacturer’s protocol, and the results are reported in NovaTech units (NTU). Values less than 9 were considered negative, those higher than 11 were considered positive, and values between 9 and 11 were considered equivocal. Patients with equivocal results were resampled and tested after 4 weeks. We considered NTU values less than 20 to be weakly positive during statistical analysis.

#### Strongyloides IgG4 RDT

The *Strongyloides* IgG4 RDT (also called SsRapid in other reports) is an advanced prototype POCT based on the lateral flow technique. It detects human anti-*Strongyloides* IgG4 in serum, plasma, or blood samples. The cassette contains a nitrocellulose membrane strip lined with a recombinant NIE (rNIE) antigen at the test line and a goat anti-mouse IgG antibody at the control line. It also has a conjugate pad impregnated with colloidal gold-conjugated anti-human IgG4 mouse monoclonal antibody. Patient serum (35 µl) is added to the bottom sample well and flows up the nitrocellulose strip. An antigen–antibody complex is formed following the binding of *Strongyloides*-specific IgG4 in the sample to the rNIE at the test line. The dried gold-conjugated anti-human IgG4 in the top oval well is then reconstituted by adding three drops of the buffer in the kit. It flows down the nitrocellulose membrane, binds to the goat anti-mouse IgG at the control line, and subsequently to the antibody–antigen complex at the test line. After 15 min, the result is interpreted as positive (indicated by observing both test and control red lines) or negative (only the control line). Scoring the test line intensity is optional; however, it is useful for research purposes. Our study visually scored positive tests from +1 to +4 on the basis of the test line intensity using a guide chart [25]. We conventionally considered +1 as weakly positive in the statistical analysis.

#### Detection of other parasitic infections

The direct smear examinations were also directed at detecting other parasites. Additionally, serology was performed using IgG ELISA commercial kits to detect antibodies to *Toxocara canis* (NovaTec Immunodiagnostica GmbH, Dietzenbach, Germany) and *Fasciola hepatica* (Pishtaz Teb, Tehran, Iran).

## Results

### Study participants

A total of 520 participants were enrolled in the study, comprising 54% (281/520) male and 46% (239/520) female participants, with a mean age of 53 years (SD 21 years). Of these, 75% (390/520) lived in urban areas, while 25% (130/520) resided in rural communities. A sizeable proportion (43.8%, 227/520) reported no underlying medical conditions. Among those with health issues, 22% (114/520) were diagnosed with cardiopulmonary diseases, 16.8% (87/520) with metabolic disorders, and 5.8% (30/520) with cancers or autoimmune diseases.

The relative peripheral blood eosinophil count (Eos %) ranged from 6% to 61%, with a mean absolute eosinophil count (AEC) of 1240 ± 892 cells/µl. The relative neutrophil count (Neut %) varied from 12% to 78%, and the mean absolute neutrophil count (ANC) was 3512 ± 1680 cells/µl. Housewives represented the largest group (184/520, 35.5%). The occupational categories included farmers/laborers (103/520, 19.9%), employees (95/520, 18.3%), and sailors (63/520, 12.2%).

On the basis of the data collected from the questionnaire, 36.6% (190/520) of the participants reported no gastrointestinal or respiratory symptoms. Among the symptomatic individuals, the most reported clinical manifestations were abdominal pain (108/520, 20.9%) and shortness of breath (112/520, 21.5%).

### Serological and coprological data

Positive ELISA results were found in 35.2% (183/520) of the participants. Six cases were classified as equivocal, and subsequent resampling and testing yielded negative results. The average NTU was 88.73 ± 23.59 in the positive and 3.33 ± 1.83 in the negative groups. Samples with optical densities higher than the ELISA’s maximum reading were considered as having an NTU of 100 in statistical calculations (*N* = 144). The IgG4 RDT was positive in 43.7% (227/520) individuals, with test line intensity scores of +1 in 7.7% (40/520), +2 in 2.7% (14/520), +3 in 5.8% (30/520), and +4 in 27.5% (143/520). Combining the results of both serological methods revealed that 44.8% (233/520) of individuals were seropositive, while 55.2% (287/520) were seronegative. Fifty IgG4 RDT-positive cases were negative by ELISA, while six patients with positive ELISA results were negative by IgG4 RDT. The kappa agreement coefficient, used to assess the concordance between the two serological methods, yielded 0.776, indicating substantial agreement. Additionally, 55.2% (287/520) of the results using the two techniques showed concordant negative, and 23.4% (121/520) exhibited maximum reaction intensity by both tests. Spearman’s test, which was used to assess the correlation between the reaction intensities of the two tests, revealed a strong positive relationship (correlation coefficient 0.772, *P* < 0.001).

Of the 373 individuals who submitted stool samples, *S. stercoralis* larvae were identified in 7.5% (28 of 373) using DS, while the APC yielded positive results in 24.9% (93 of 373). In total, 95 cases were copro-positive while 278 were copro-negative.

Among the copro-positive cases, 26 used both parasitological methods, while two samples were positive by DS but negative by APC. Additionally, 67 cases were positive by APC but negative by DS (*κ* = 0.355, *P* = 0.055). All copro-positive individuals showed strong positive results in the ELISA, except for one ELISA-negative case that was positive by IgG4 RDT (+4). The IgG4 RDT showed strong positivity for all copro-positive individuals, except for one case with a weakly positive result (+ 1) but that was strongly ELISA-positive. Overall, all copro-positive cases demonstrated at least one strongly positive serological result.

Two hundred participants were negative for all serological and coprological assessments. Among individuals with negative coprological results, 44 were strongly positive in both serological tests. There were also 34 cases with negative coprological results, but they were positive (albeit with a weak reaction) in one of the serological tests. Overall, the kappa coefficient of agreement between the serological and coprological methods was 0.566 (*P* = 0.039).

A significant association was observed between copro-positive cases and several variables, including age group, literacy level, occupation, soil contact, and gastrointestinal and respiratory symptoms (Table 1). There were 30 immunocompromised participants—with cancer or autoimmune disorders. Of these 30 participants, 10 were seropositive for *Strongyloides* and 8 were copro-positive. All copro-positive cases in this study were treated by an infectious disease specialist with albendazole (the only anti-*Strongyloides* medication available in the region), 400 mg orally two times a day for 7 days.

**Table 1:** Demographic and clinical data of the study participants based on coprological methods

Variable	Group	Copro-positive (*N* = 95)	Copro-negative (*N* = 278)	*P*-value
Sex	Male	55.8	56.5	0.907
	Female	44.2	43.5	
Age	< 20 years	1.1	19.8	0.001
	20–40 years	0.0	25.9	
	41–60 years	22.1	22.3	
	> 60 years	76.8	32	
Residency	Urban	78.3	75.3	0.579
	Rural	21/7	24.7	
Literacy	Illiterate	54.3	29.9	0.001
	Elementary	32.6	23.1	
	Literate	13.1	47.1	
Occupation	Farmer	25	18	0.001
	Employee	4.3	26.7	
	Sailor	20.7	7.8	
	Housewife	40.2	29.5	
	Other	9.8	18	
Gastrointestinal symptoms	Asymptomatic	31.5	56.1	0.001
	Abdominal pain	34.8	15.8	
	Diarrhea	9.8	5.4	
	Constipation	16.3	17.2	
	Other	7.6	5.4	
Pulmonary symptoms	Asymptomatic	67.4	75	0.014
	Cough	9.8	3.2	
	Dyspnea	21.7	15.9	
	Other	1.1	5.9	
Underlying diseases	Without disease	38	49.5	0.040
	Metabolic	25	14.1	
	Cardiopulmonary	17.4	20.9	
	Cancerous/autoimmune	8.7	3.6	
	Other	10.9	11.8	
Soil contact	Yes	77.2	38.8	0.001
	No	22.8	61.2	

The percentages of gastrointestinal and respiratory symptoms were calculated separately within their respective categories. For example, the percentage of abdominal pain was relative to all gastrointestinal symptoms, not the total cohort

Regarding the detection of other parasites, of the 373 examined stool samples, *Enterobius vermicularis* ova was found in 2 samples (0.53%), *Entamoeba histolytica*/*dispar* cysts in 6 samples (1.6%), *Giardia* cysts in 9 samples (2.41%), and vacuolar forms of *Blastocystis hominis* in 14 (3.75%) samples. Additionally, on the basis of serology, *Toxocara* antibodies were detected in four samples (0.77%, *n* = 520), and there was no *Fasciola hepatica*-positive samples. Six of the above 35 other parasite-positive samples were also positive for *Strongyloides* larvae (by coprological analysis) and both serological assays. Thus, only 4 samples from 29 larvae-negative individuals were positive by the serological tests. They comprised three  *B. hominis*-positive samples, which two were positive by both serological assays, and one other *B. hominis* samples was positive by only the IgG4 RDT. One *Toxocara* antibody-positive sample was also positive by the IgG RDT.

### Evaluation the performance of diagnostic tests

On the basis of the traditional coprological reference (TCR), which mandates at least one positive coprological test result, 95 “true positive” cases and 278 “true negative” cases were identified. The receiver operating characteristic (ROC) curve analysis indicated that the AUC of the ELISA was 0.908 (95% confidence interval (CI) 0.878–0.939). The IgG4 RDT demonstrated an AUC of 0.867 (95% CI 0.832–0.902). When the results of both serological methods were combined, the AUC was 0.860 (95% CI 0.824–0.896).

**Fig. 1:**
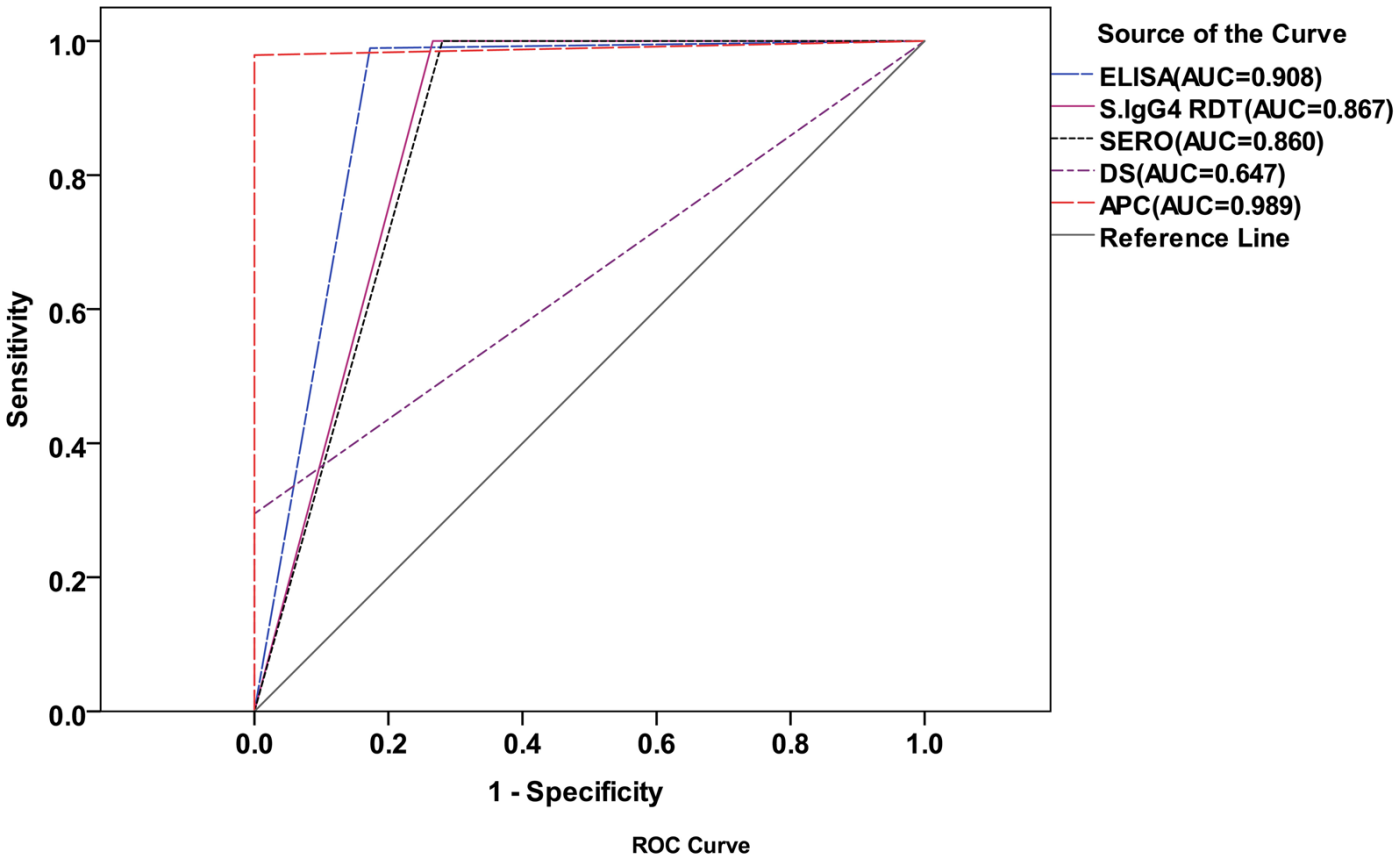
ROC curve analysis of diagnostic methods versus traditional coprological reference (TCR) standard

Both serological tests revealed comparable high sensitivity and negative predictive values; however, the specificity of the ELISA (82.7%) exceeded that of the IgG4 RDT (73.4%) (Table 2). Among all methods evaluated, the DS yielded the lowest AUC of 0.647 (95% CI 0.576–0.719) and demonstrated the least diagnostic sensitivity (29.5%) (Fig. 1; Table 2).

**Table 2:** Sensitivity, specificity, PPV, NPV, and *κ* value based on the TCR (*N* = 373) and CRS (*N* = 520)

Reference standard	Test	Sensitivity (%)	Specificity (%)	PPV	NPV	Kappa
TCR	RDT	100	73.4	56.2	100	0.584
	ELISA	98.9	82.7	66.2	99.6	0.702
	Sero	100	71.9	54.9	100	0.566
	DS	29.5	100	100	80.57	0.384
	APC	97.9	100	100	99.28	0.986
CRS	RDT	98.5	89	84.1	99	0.845
	ELISA	92.8	99.1	98.4	95.8	0.929
	Sero	100	88	83.3	100	0.846
	DS	9.2	100	100	65.8	0.224
	APC	63.7	100	100	81.1	0.681
	COPRO	65.1	100	100	81.7	0.694

*PPV* positive predictive value, *NPV* negative predictive value, *RDT* IgG4 rapid diagnostic test, *ELISA* enzyme-linked immunosorbent assay, *Sero* serology, *DS* direct smear, *APC* agar plate culture, *TCR* traditional coprological reference, *CRS* combination of coprology and serology methods, *COPRO* combination of coprology methods

**Fig. 2:**
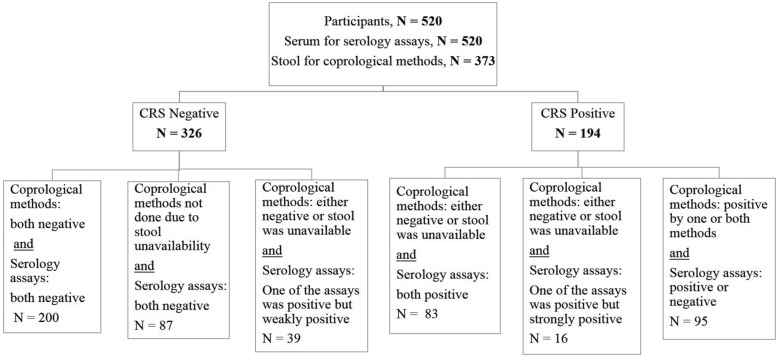
Flowchart of studied samples based on the described composite reference standard

On the basis of the composite reference standard (CRS) outlined in Fig. 2, 194 individuals were classified as positive and 326 as negative. Cohen’s *κ* coefficient of agreement between the CRS and TCR was calculated to be 0.694, indicating substantial agreement between the two reference methods.

**Fig. 3:**
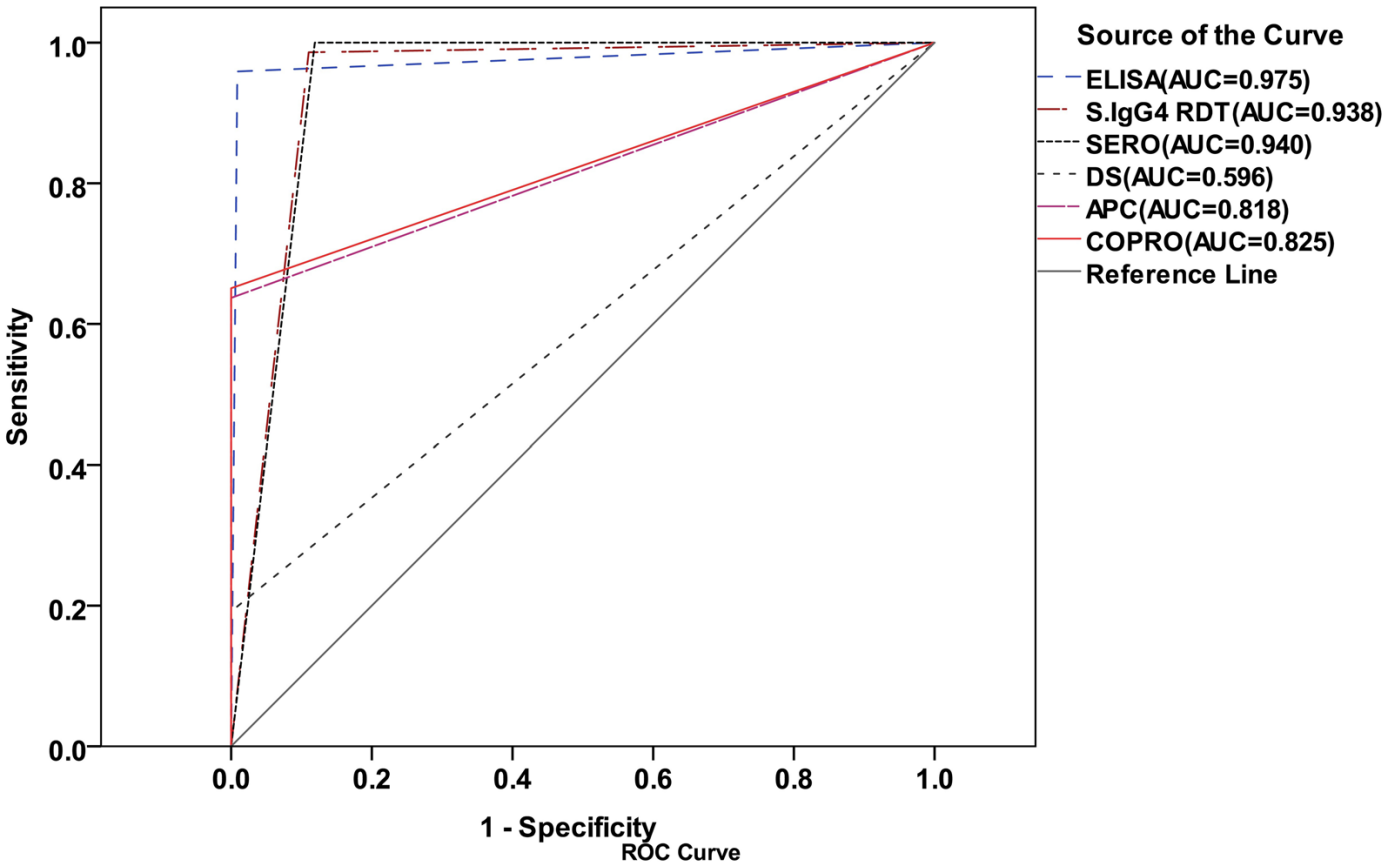
ROC curves showing the area under the curve (AUC) of all used diagnostic methods compared with the composite reference standard (CRS)

Using the CRS, the AUC of the ELISA, IgG4 RDT, APC, and DS was 0.975, 0.938, 0.818, and 0.596, respectively, and 0.940 for the combination of serological methods. Meanwhile, the combination of coprological methods yielded an area under the curve (AUC) of 0.825 (Fig. 3).

The IgG4 RDT demonstrated the highest diagnostic sensitivity at 98.5%, whereas the DS displayed the lowest sensitivity at 9.2%. Notably, the combined sensitivity of the two serological methods reached 100%, while the combination of coprological methods achieved a sensitivity of 63.7%.

When comparing the serological methods against the CRS, the IgG4 RDT indicated a higher sensitivity but lower specificity than the ELISA. Also, when evaluating the coprological methods against the CRS, the sensitivity of the direct smear method was seven times lower than that of the APC (Table 2).

### Eosinophil count

Among the copro-positive cases, the mean Eos % was 20.48 ± 7.73% with an AEC of 1648 ± 749 cells/µl. In contrast, the copro-negative cases showed a mean Eos % of 15.22 ± 6.85% with an average AEC of 1166 ± 1023 cells/µl. The Mann–Whitney *U* test demonstrated a statistically significant difference in eosinophil counts between the copro-positive and copro-negative groups (*U* = 6823, *P* < 0.001). The mean eosinophil counts for seropositive cases were 17.91 ± 6.90% for Eos % and 1393 ± 723 for AEC. In comparison, seronegative individuals had mean values of 14.70 ± 6.59% and 1116 ± 993, respectively. The differences between these two groups were also statistically significant (*U* = 22,168, *P* < 0.001).

ROC curve analysis was performed to evaluate the diagnostic performance of the eosinophilia index in detecting infection. The AUC values were 0.741 and 0.701 for TCR and CRS, respectively, demonstrating that the eosinophilia index was moderately accurate in identifying infected individuals.

### Post-treatment follow-up

Follow-up assessments were conducted on 30 patients at a minimum of 3 months post-treatment. The overall results demonstrated a significant reduction (*P* < 0.001) in both the Eos % and AEC following treatment, with Eos % decreasing from 21.87 ± 7.85% to 8.30 ± 6.97%, and AEC decreasing from 1642 ± 698 cells/µl to 608 ± 603 cells/µl. The detailed results are provided in the table in the Supplementary Information.

Five patients (17%) remained copro-positive after treatment, and their serological results were still strongly positive. Among these, one patient tested positive via both coprological methods, while four patients were positive only by the APC. Three patients were strongly positive by the ELISA and the IgG4 RDT despite being negative by coprology.

Concerning the serological tests, 73% (22 of 30) of the post-treatment patients’ samples remained ELISA-positive, while 90% (27 of 30) tested positive by the IgG4 RDT. The average NTU values of the ELISA significantly decreased from 98.15 ± 10.11 to 37.59 ± 36.21, and intensity scores in the IgG4 RDT showed a reduction in 21 patients (70%).

**Fig. 4:**
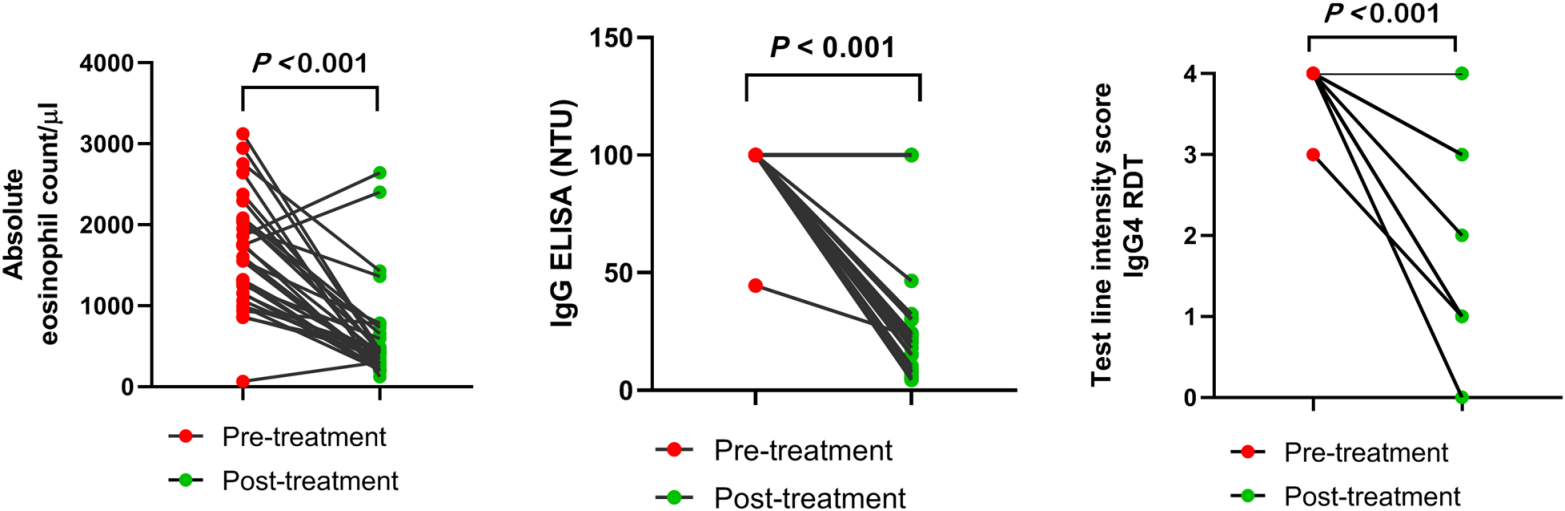
Comparison of 30 pre- and post-treatment patient samples. **A.** absolute eosinophil count (AEC, cells/µl blood). **B.** IgG ELISA (NTU). **C.** IgG4 RDT test line intensity scores. The number of lines C seems fewer owing to the overlapping data points. The Supplementary Information includes a table with more details of the results of the 30 samples

Statistical analysis using the Wilcoxon test indicated a significant difference in the results before and after treatment (Fig. 4) (*P* < 0.001). However, the Spearman correlation test revealed a weak correlation between the intensity values of the ELISA and IgG4 RDT results, with a correlation coefficient of 0.224 (*P* = 0.233).

## Discussion

The findings of this study shed light on a frequently overlooked issue: chronic strongyloidiasis, particularly in hypoendemic regions. A 2021 report on a study in Khuzestan showed a coprological prevalence of 2.7% and a serological prevalence of 8.7% in an immunocompromised population [26]. However, the present study among a specific eosinophilic population in the same region showed high rates of positive coprology (95/373, 25.5%) and serology (233/520, 44.8%). A noteworthy finding is the high percentage of coprological (8/30) and seropositive cases (10/30) detected among immunocompromised participants, underscoring the urgent need for targeted monitoring and treatment strategies in this vulnerable population.

The four techniques employed in this study produced varying detection frequencies, indicating that none fully and accurately accomplished individual diagnosis when used in isolation [27]. Thus, despite various coprological, serological, and molecular techniques for diagnosing strongyloidiasis, none can reliably distinguish between healthy individuals and those affected by the disease [9]. Therefore, using more than one diagnostic method is encouraged to detect *Strongyloides* infection [28].

The absence of a dependable “gold standard” for diagnosis significantly hinders the detection of this infection and our understanding of the global burden of strongyloidiasis. This challenge complicates not only individual diagnoses but also treatment follow-up and the evaluation of new diagnostic methods. Therefore, it is essential to select an appropriate diagnostic method(s) or algorithm that is tailored to the specific characteristics of the study group, the objectives of the research, the capabilities of the staff, ease of implementation, the potential consequences of misdiagnosis, and the necessary balance between sensitivity and specificity [29].

Regarding the TCR, the sensitivity of the APC was 3.3 times greater than DS. This is consistent with previous studies that reported a sensitivity for APC that was 1.6–6 times that of DS [8]. The ROC curve analysis also revealed a significant difference in the diagnostic power of the two tests, with AUC values of 0.989 for the APC test and 0.647 for the DS method. Notably, the DS-positive but APC-negative cases highlighted the potential impact of factors such as dead larvae in stool samples (due to improper storage/transport, medication effects, or immune system interactions) on diagnostic accuracy. Furthermore, given that larvae are excreted intermittently, a negative culture result based on a single stool sample cannot reliably indicate the absence of infection. The US Centers for Disease Control and Prevention (CDC) recommends conducting seven stool examinations to rule out infection, and studies have emphasized that a three-step culture method is crucial for attaining the highest diagnostic performance [13, 27]. As a result, collecting serial stool samples in the present study would have improved the coprological detection rate observed, significantly narrowing the gap between the coprological and serological frequencies. However, multiple samplings of an individual in a population study are impractical.

Despite the high sensitivities of the IgG4 RDT and ELISA (100% and 98.9%, respectively) compared with the TCR method, their specificities were lower, at 73.4% and 82.7%, respectively (Table 2). The sensitive serological assays may identify *Strongyloides* infection with very low larvae output that is undetectable by the TCR methods. Such scenarios can artificially lower the calculated diagnostic specificity of the serological assays, making it appear poorer than it is [5, 8, 30].

On the basis of the serological results with samples containing co-infections with other parasites, there does not seem to be a significant cross-reaction issue; this may be due to the use of recombinant antigens in both serology tests. Moreover, several reports on the IgG4 RDT have shown it to be highly specific [25, 31, 32]. Nevertheless, since there were many patients positive by serology but negative by coprological assays in this study, and the general fact that cross-reaction remains a common confounding factor in serological assays, one should always be alert to any potential cross-reactivity issue.

Clinical-based studies such as the present study face challenges in accurately classifying individuals as seropositive and copro-negative, particularly those who do not provide stool samples [4]. Integrating various imperfect methods as a composite reference standard (CRS) can help clarify disease status and enhance clinical decision-making for treating affected individuals. The main goal is to identify cases that necessitate clinical intervention rather than simply determining the presence or absence of disease. This flexible approach enables researchers to improve the sensitivity or specificity of the final CRS by choosing between the OR rule (any positive case) or the AND rule (all positive cases) when integrating methods, depending on the objectives of the study [33, 34]. The aim is to ensure that no potential positive cases are overlooked. To achieve this, we drew upon the insights gained from the various cases in the present study, as well as the experiences of other researchers [30].

With the new CRS encompassing all participants, the number of positive cases identified rose from 95 to 194. While the CRS proved effective in detecting latent and very mild cases of infection, we acknowledge the potential for false positives. However, given the risks associated with the progression of autoinfection to hyperinfection and the high tolerability of the first-line treatment (ivermectin), which has minimal side effects, it seems prudent to treat all individuals who test positive [30]. Using the CRS criterion, the AUC for the DS technique is 0.59, with a sensitivity seven times lower than that of the APC method. This notable disparity highlights the considerable limitations of the DS technique in identifying mild and chronic infections. Therefore, CRS must integrate more sensitive coprological or serological techniques to improve diagnostic accuracy and ensure comprehensive patient evaluation [14, 35]. According to the same reference, both ELISA and IgG4 RDT indicated an AUC exceeding 0.9, signifying their remarkable diagnostic efficacy. Additionally, the overall negative predictive value (NPV) of serological methods ranged from 95.8% to 100%, aligning with findings from previous studies [5, 14]. Consequently, when the objective is population screening, serological methods emerge as the optimal choice for detecting infections [36].

ELISA, the serological test most widely utilized for diagnosing *S. stercoralis* infections, presents several advantages, such as high sensitivity and throughput [37]. However, its reliance on a cold chain for kit transport, the necessity of sending samples to a central laboratory, and the requirement for trained personnel significantly limit its applicability in field settings [38]. Developing ELISA as a POCT could address these limitations, providing a user-friendly, cost-effective, and rapid solution tailored for field conditions and low-income endemic communities [25]. The IgG4 RDT showed robust diagnostic indices comparable to the ELISA. The assessment revealed a kappa coefficient of 0.776, indicating substantial agreement and a strong correlation, as evidenced by the Spearman coefficient (*ρ* = 0.772; *P* < 0.001). These results highlight the potential of the IgG4 RDT as a viable alternative to ELISA, enabling infection diagnosis in targeted areas without requiring specialized equipment, extensive logistical support, or trained laboratory personnel.

The results of this study indicated that the IgG4 RDT showed slightly higher sensitivity but lower specificity when compared with the ELISA, as evaluated by both reference standards. These discrepancies are likely due to variations in the detected antibody subclasses, differences in the structural characteristics of the testing methodologies, and the specific types of antigens employed in their design. Notably, IgG1 is recognized as an effective marker for the early stages of infection, while IgG4 is crucial as a secreted subclass in chronic parasitic infections, such as strongyloidiasis [13, 39]. This distinction in antibody subclasses can significantly impact the diagnostic performance of the tests [40, 41]. Notably, our study found that 76.8% (73 out of 95) of copro-positive cases and 63.94% (149 out of 233) of seropositive cases were in the elderly population. This indicates that the IgG4 RDT might be more sensitive than ELISA in detecting infections within the demographic of this study. Previous studies evaluating the diagnostic accuracy of the IgG4 RDT have reported sensitivities ranging from 82.4% to 97% and specificities from 59.5% to 100% [25, 31, 32, 42–44]. Several key factors contribute to the variability in these results, including the geographical regions and their parasite diversity, the types of samples tested (serum, plasma, and fingerstick blood), the study type (laboratory-based with serum bank samples or field studies), the level of *Strongyloides* endemicity in the field studies, and the reference methods used (TCR or CRS). Generally, studies employing combined reference methods tend to demonstrate higher sensitivity and specificity. In contrast, studies utilizing standard coprological methods and those relying on field sampling instead of serum banks often report lower specificity [31].

Unexplained elevation of eosinophilia is a crucial laboratory finding. It is often the sole diagnostic indicator for strongyloidiasis [38, 40]. Eosinophilia occurs in 50–75% of chronic asymptomatic or symptomatic patients owing to tissue migration of filariform larvae during cycles of reinfection and to adult worms in the mucosa and/or submucosa causing tissue irritation [8, 45, 46]. In an experimental study involving cell-deficient and antibody-depletion mice infected with *Strongyloides ratti*, it was demonstrated that eosinophils are involved in trapping and eliminating L3 in the skin and tissues [47], as well as in parasite ejection from the intestine [48]. Nevertheless, it must be emphasized that strongyloidiasis can occur without eosinophilia. Severe and complicated forms such as disseminated strongyloidiasis are often associated with a normal absolute eosinophil count [49].

Our results revealed significant differences in eosinophil counts between the participants who were negative by serological and coprological tests and those who were positive by either serological and/or coprological tests (*P* < 0.001). Moreover, compared with the TCR, the AUC of 0.741 for the eosinophilia index highlights its relatively good diagnostic capability for identifying infected individuals [38, 50]. Eosinophil counts can sometimes fluctuate in chronic strongyloidiasis. Previous studies have reported sensitivity rates for this biomarker ranging from 60.9% to 82% and specificity rates between 71.1% and 96% [38, 50]. In our study, among the 34 patients with an absolute eosinophil count below 500 cells/µl blood, we identified five seropositive cases, one of which was coprology positive, further supporting this observation.

Previous research studies indicated that approximately half of the patients with strongyloidiasis were asymptomatic, while the other half presented with at least one nonspecific symptom, including skin manifestations, abdominal pain, diarrhea, cough, or dyspnea [46, 51]. In the present study, 36.34% (189/520) reported no gastrointestinal or respiratory complaints. Among those who were symptomatic, the common manifestations included abdominal pain, constipation, diarrhea, shortness of breath, and cough. The diverse and nonspecific clinical presentation of chronic strongyloidiasis presents significant challenges for accurate diagnosis and screening when relying solely on symptomatology. While common clinical symptoms and risk factors, such as occupation, education level, age, and history of soil contact, can heighten suspicion, the absence of these factors or symptoms should not rule out the possibility of infection or exclude individuals from being screened [52–54].

In the follow-up phase of the study, all patients who experienced treatment failure were part of the group that had been treated for over a year. They included five cases with positive coprology and three with strong serological concordance. While the inadequate efficacy of albendazole (400 mg orally two times a day for 1 week) as a second-line treatment [55, 56] is a concern in this study, the possibility of reinfection cannot be dismissed. The Wilcoxon test indicated a significant reduction in the intensity of reactions for both the ELISA and IgG4 RDT, with this trend becoming more pronounced over time. Researchers emphasize monitoring the declining antibody trend to ensure treatment success [5, 8]. For cases where the pre- and post-treatment samples showed the same IgG4 RDT line intensity scores and ELISA NTUs, performing serum dilutions before testing the paired samples could help elucidate whether there is a reduction in antibody titers, which would signal a positive treatment outcome.

The IgG4 RDT is a suitable alternative to ELISA owing to its convenience and speed. Comparing the pre- and post-treatment phases, a weak correlation was observed between the intensity of the IgG4 RDT and ELISA reactions, which may suggest an asymmetric decrease due to the differing half-lives of the IgG subclasses [57].

Concurrently, the Wilcoxon test results revealed a significant decrease in eosinophil cell counts, returning to normal in patients who underwent successful treatment (*P* < 0.1). Other studies have also reported similar findings [46, 58]. Although, in general, AEC cannot be taken as a marker for treatment success since it intrinsically fluctuates in strongyloidiasis, its persistence is suggestive of treatment failure [46, 59].

The main limitation of this study was the reliance on a single stool sample for coprological diagnosis. Owing to the low and intermittent *Strongyloides* larvae output in the stool, multiple samples on different days would have increased the diagnostic sensitivity of the coprological tests. Owing to poor compliance, the second limitation was that only 30 larvae-positive individuals were followed up instead of all the larvae-positive cases (*n* = 95). The third limitation is the use of albendazole for treatment instead of ivermectin, the first-line drug for strongyloidiasis. If ivermectin had been used, it may have resulted in better larval clearance rate and faster antibody level reduction. Finally, as mentioned above, in cases where the RDT test line intensity scores or the ELISA NTUs were the same before and after treatment, testing serially diluted samples would have been useful to show the antibody titer decline. However, this was not performed owing to insufficient rapid test and ELISA kits.

## Conclusions

This study shows a high prevalence of *Strongyloides* infection among the eosinophilic patient population. The *Strongyloides* IgG4 RDT could be a useful test for diagnosing strongyloidiasis in endemic areas, with a remarkable sensitivity of 98.5%. Its user-friendliness and nonrequirement for cold-chain transportation make it ideal for screening vulnerable populations. Nevertheless, ongoing local assessments and further validation are essential to refine its performance across various contexts.

## Supplementary Information

Additional file 1. S1. Absolute eosinophil count (µl), eosinophil percentage, coprological and serological data of 30 infected individuals pre- and post-treatment.

## Data Availability

Data supporting the main conclusions of this study are included in the manuscript..

## Abbreviations

*S*. *stercoralis*: *Strongyloides stercoralis*
APC: Agar plate culture
ELISA: Enzyme-linked immunosorbent assay
POCT: Point-of-care test
WHO: World Health Organization
IgG4: Immunoglobulin G4
CBC: Complete blood count
SD: Standard deviation
CI: Confidence intervals
ROC: Receiver operating characteristic
κ: Cohen’s kappa coefficient
DS: Direct stool examination
IgM: Immunoglobulin M
NTU: NovaTech units
µl: Microliter
Eos: Eosinophil
AEC: Absolute eosinophil count
ANC: Absolute neutrophil count
TCR: Traditional coprological reference
AUC: Area under the curve
CDC: Centers for Disease Control and Prevention
NPV: Negative predictive value
PPV: Positive predictive value
