# Supplementary material for: Strongyloides stercoralis prevalence and diagnostic efficacy of an IgG4 rapid test in an eosinophilic population in Khuzestan Province, southwestern Iran
Source: Parasit Vectors. 2025 Jul 22;18:291. doi: 10.1186/s13071-025-06910-z (PMC12281991; doi:10.1186/s13071-025-06910-z)
Supplement: Supplementary file 1 — Additional file 1. S1. Absolute eosinophil count (µl), eosinophil percentage, coprological and serological data of 30 infected individuals pre- and post-treatment. [file 13071_2025_6910_MOESM1_ESM.docx]

S1.

| Patient | Time of examination | Interval between two stages | AEC cell/µl | | Eosinophil % | | Coprological and serological tests | | | | | |
| --- | --- | --- | --- | --- | --- | --- | --- | --- | --- | --- | --- | --- |
|  |  |  | **Pretreatment** | **Posttreatment** | **Pretreatment** | **Posttreatment** | **Pretreatment** | | | **Posttreatment** | | |
|  | **Pretreatment** | **Month** |  |  |  |  | **DS*/ APC*** | **ELISA*** | **RDT*** | **DS/ APC** | **ELISA** | **RDT** |
|  | **Posttreatment** |  |  |  |  |  |  |  |  |  |  |  |
| 1 | 2024/02/29  2024/08/02 | 5 | 992 | 486 | 16 | 6 | Neg*/Pos* | OVER | 4+ | Neg/Neg | 46.51 | 3+ |
| 2 | 2024/01/21  2024/07/29 | 6 | 2945 | 432 | 31 | 6 | Pos/Neg | OVER | 4+ | Neg/Neg | 14.88 | 1+ |
| 3 | 2023/12/11  2024/07/29 | 7 | 1738 | 408 | 22 | 6 | Neg/Pos | OVER | 4+ | Neg/Neg | 23.71 | 3+ |
| 4 | 2023/08/18  2024/07/29 | 11 | 3120 | 444 | 30 | 6 | Neg/Pos | OVER | 4+ | Neg/Neg | 32.48 | 3+ |
| 5 | 2024/03/06  2024/07/31 | 4 | 2080 | 728 | 26 | 8 | Neg/Pos | OVER | 4+ | Neg/Neg | 22.46 | 2+ |
| 6 | 2024/04/25  2024/08/01 | 3 | 1552 | 248 | 16 | 4 | Neg/Pos | OVER | 4+ | Neg/Neg | 23.43 | 1+ |
| 7 | 2024/02/29  2024/06/21 | 3 | 864 | 408 | 18 | 6 | Neg/Pos | OVER | 4+ | Neg/Neg | 30.04 | 1+ |
| 8 | 2024/02/25  2024/07/30 | 5 | 1314 | 395 | 18 | 5 | Neg/Pos | OVER | 4+ | Neg/Neg | 20.92 | 1+ |
| 9 | 2023/08/09  2024/08/31 | 12 | 990 | 208 | 15 | 4 | Neg/Pos | OVER | 4+ | Neg/Neg | 30.55 | 3+ |
| 10 | 2022/12/06  2024/05/19 | 17 | 1248 | 594 | 26 | 11 | Neg/Pos | OVER | 4+ | Pos/Pos | OVER | 4+ |
| 11 | 2024/02/09  2024/07/05 | 4 | 1760 | 192 | 40 | 4 | Neg/Pos | OVER | 4+ | Neg/Neg | 15.44 | 4+ |
| 12 | 2024/01/08  2024/04/07 | 3 | 2640 | 122 | 30 | 1 | Neg/Pos | OVER | 4+ | Neg/Neg | 7.32 | 1+ |
| 13 | 2020/03/02  2024/07/11 | 52 | 1950 | 1361 | 25 | 17 | Neg/Pos | OVER | 4+ | Neg/Pos | OVER | 4+ |
| 14 | 2021/12/12  2023/09/26 | 21 | 1550 | 784 | 25 | 14 | Neg/Pos | OVER | 4+ | Neg/Neg | OVER | 4+ |
| 15 | 2020/01/05  2022/08/20 | 31 | 2376 | 425 | 27 | 5 | Pos/Pos | OVER | 4+ | Neg/Neg | 5.03 | Neg |
| 16 | 2024/03/02  2024/07/28 | 4 | 936 | 774 | 12 | 9 | Pos/Pos | 44.57 | 3+ | Neg/Neg | 22.89 | 1+ |
| 17 | 2023/02/09  2024/01/06 | 11 | 1320 | 189 | 20 | 3 | Neg/Pos | OVER | 4+ | Neg/Neg | 9.13 | 1+ |
| 18 | 2021/11/22  2024/05/16 | 29 | 1863 | 2640 | 27 | 33 | Pos/Pos | OVER | 4+ | Neg/Pos | OVER | 4+ |
| 19 | 2023/02/22  2024/07/28 | 17 | 1062 | 330 | 9 | 3 | Pos/Pos | OVER | 4+ | Neg/Neg | 5.85 | Neg |
| 20 | 2024/03/16  2024/08/04 | 4 | 2295 | 280 | 26 | 5 | Neg/Pos | OVER | 4+ | Neg/Neg | 23.77 | 4+ |
| 21 | 2018/07/01  2023/11/08 | 64 | 66 | 304 | 2 | 4 | Pos/Pos | OVER | 4+ | Neg/Pos | OVER | 4+ |
| 22 | 2020/02/21  2024/05/13 | 50 | 1748 | 2400 | 23 | 25 | Neg/Pos | OVER | 4+ | Neg/Pos | OVER | 4+ |
| 23 | 2024/01/13  2024/07/28 | 6 | 1280 | 216 | 16 | 4 | Neg/Pos | OVER | 4+ | Neg/Neg | 10.49 | 2+ |
| 24 | 2022/03/17  2024/07/02 | 27 | 2030 | 654 | 35 | 15 | Neg/Pos | OVER | 4+ | Neg/Neg | 7.71 | 1+ |
| 25 | 2022/11/17  2023/06/21 | 6 | 1600 | 284 | 20 | 4 | Neg/Pos | OVER | 4+ | Neg/Neg | 24.44 | 3+ |
| 26 | 2022/04/01  2023/10/12 | 18 | 1156 | 208 | 17 | 4 | Pos/Pos | OVER | 4+ | Neg/Neg | 18.05 | 1+ |
| 27 | 2022/12/26  2024/07/18 | 18 | 855 | 486 | 15 | 6 | Neg/Pos | OVER | 3+ | Neg/Neg | 20.15 | 1+ |
| 28 | 2021/01/29  2023/08/19 | 30 | 2052 | 468 | 27 | 12 | Neg/Pos | OVER | 4+ | Neg/Neg | 4.38 | Neg |
| 29 | 2021/08/14  2023/10/23 | 26 | 2750 | 1428 | 22 | 14 | Neg/Pos | OVEW | 4+ | Neg/Neg | 8.20 | 3+ |
| 30 | 2022/04/17  2023/08/08 | 15 | 1140 | 360 | 20 | 5 | Neg/Pos | OVER | 4+ | Neg/Neg | OVER | 4+ |
